# URINARY INCONTINENCE IN US ADULTS WITH DIABETES WHO ARE GUIDELINE-RECOMMENDED TO RECEIVE SGLT2IS: NHANES 2013–2020

**DOI:** 10.1093/geroni/igae098.1455

**Published:** 2024-12-31

**Authors:** Alexandra Lee, Sei Lee

**Affiliations:** University of California San Francisco, San Francisco, California, United States; University of California San Francisco, San Francisco, California, United States

## Abstract

Sodium-glucose cotransporter-2 inhibitors (SGLT2is) are now recommended as first-line cardiorenal protective therapy for many adults with type 2 diabetes but are also associated with increased risk of urogenital infections. While urinary incontinence is common among older adults and increases the risk of urogenital infections, there is limited data on the prevalence of incontinence among adults with diabetes, particularly those eligible to receive SGLT2is. We included participants with diabetes from the nationally-representative National Health And Nutrition Examination Survey (NHANES) 2013-2020. We determined whether each participant would be ADA guideline-recommended to receive SGLT2is (a) due to heart failure or chronic kidney disease, or (b) due to atherosclerotic cardiovascular disease or high cardiovascular risk (in which case they could also alternatively be treated with GLP-1RAs). We defined frequent incontinence as self-reported leaking urine “daily/nightly” or “several times per week.” There were 2,366 NHANES participants with type 2 diabetes. Among adults aged 55-59, 14.9% were guideline-recommended an SGLT2i, 22.7% of whom had frequent incontinence; among adults aged 70+, 27.0% were recommended to receive an SGLT2i, 41.0% of whom had frequent incontinence. Among adults aged 55-59, 54.6% were guideline-recommended either an SGLT2i or GLP-1RA, 20.6% of whom had frequent incontinence; among adults aged 70+, 45.1% were recommended to receive either an SGLT2i or GLP-1RA, 34.9% of whom had frequent incontinence. Prescribers should use caution for patients with incontinence who are initiating SGLT2is. While urogenital infections are typically not life-threatening, they can significantly impact quality of life and may not be aligned with health goals.

